# Concentrations and Probabilistic Health Risks of Seven Metals in Face and Eye Cosmetics Across Seven Asian Countries

**DOI:** 10.3390/toxics14020167

**Published:** 2026-02-11

**Authors:** Sohyeon Choi, Jae-Hyun Kim, Aram Lee, Yong-Jun Jeon, Won Kim, Inja Choi, Jeongim Park

**Affiliations:** 1 Department of Environmental Health Sciences, Soonchunhyang University, Asan 31548, Republic of Korea; sohyeon9535@gmail.com (S.C.); kjh980617@gmail.com (J.-H.K.); lar2654@naver.com (A.L.); jyjg510@gmail.com (Y.-J.J.); 2 Wonjin Institute for Occupational and Environmental Health, Seoul 02221, Republic of Korea; gganna@hanmail.net (W.K.); spannerchoi@wioeh.org (I.C.)

**Keywords:** heavy metals, mercury adulteration, cosmetic safety, source apportionment, probabilistic risk assessment

## Abstract

Despite global restrictions like the Minamata Convention, heavy metal contamination in cosmetics remains a critical public health concern, with limited cross-country comparative data on heavy metal concentrations in cosmetics across Asian markets. We measured Hg, Pb, As, Cd, Sb, Cr, and Ni contents in 189 cosmetic products purchased in 2022 in Bangladesh, India, Indonesia, Korea, Malaysia, the Philippines, and Vietnam. Samples were screened by handheld X-ray fluorescence; Hg was quantified by a direct mercury analyzer and As, Cd, Cr, Ni, Pb, and Sb were quantified by ICP-OES. Principal component analysis (PCA) was used to characterize metal co-occurrence patterns, and Monte Carlo simulation was applied to estimate dermal systemic exposure dose, hazard quotients (HQ), and lifetime cancer risk (LCR). Mercury in face creams exhibited extreme heterogeneity (range: ND-67,000 mg/kg), while eye cosmetics contained elevated Arsenic levels (median 4.13 mg/kg). PCA distinctively separated Hg (PC2) from geogenic metals (As/Cr/Ni on PC1), suggesting intentional adulteration. Probabilistic risk estimates indicated upper-tail non-cancer risk for Hg in facial creams (95th percentile HQ 6.32; P[HQ>1] = 24.4%). As produced the highest LCR estimates (facial cream 95th percentile 2.60 × 10^−4^). These findings indicate product-type-specific metal patterns and highlight a subset of facial products with extreme Hg levels that can drive substantial upper-percentile risk, supporting the need for targeted market surveillance and enforcement.

## 1. Introduction

Cosmetics have become an integral part of daily life, with the global market expanding rapidly. However, the safety of these products remains a contentious public health issue due to the persistent presence of toxic heavy metals. Metals such as lead (Pb), cadmium (Cd), and arsenic (As) are typically considered unintentional impurities derived from mineral raw materials like mica or talc [1,2]. In contrast, mercury (Hg) represents a unique threat; despite global prohibitions like the Minamata Convention (2013), it continues to be illegally added to skin-lightening creams due to its potent ability to inhibit tyrosinase enzymes and suppress melanogenesis [3,4].

Previous monitoring studies have reported sporadic violations of safety standards in the Asian market. For instance, Akhtar et al. (2022) detected extreme mercury concentrations in products sold online in Southeast Asia, attributing it to weak post-market surveillance [5]. However, most existing literature has focused primarily on identifying non-compliance concentrations, often lacking a robust statistical distinction between “incidental contamination” and “intentional adulteration.” Furthermore, while the risks of classic heavy metals are well-documented, emerging contaminants like Antimony (Sb)—often associated with eye cosmetics and traditional eyeliners (e.g., *Kohl or Surma*)—have received limited attention in the context of modern commercial cosmetics [6]. Given that the eyelid dermis is thin and susceptible to absorption, the lack of data on antimony in the Asian market constitutes a significant knowledge gap.

Another critical limitation in previous studies lies in the methodology of health risk assessment. Traditional assessments, such as the comprehensive study by Lim (2018) [7], typically employ a deterministic approach relying on mean or maximum concentrations to estimate consumer risk. While this method is effective for regulated markets with consistent product quality, it often fails to capture the variability of exposure in markets plagued by illicit adulteration. Specifically, when data distribution is highly skewed by extreme outliers (e.g., >10,000 mg/kg), the deterministic averaging method risks significantly underestimating the potential hazard to heavy users or the high-risk upper percentile of the population. To address this, a probabilistic approach is required to reflect the full range of realistic exposure scenarios.

Heavy metals present in cosmetic products can result in repeated dermal contact and, depending on metal species, formulation, and skin condition, may contribute to percutaneous uptake and local or systemic exposure [2,8]. Across metals, several toxicological endpoints are shared and are particularly relevant from a co-exposure perspective, including oxidative stress and redox imbalance, inflammatory signaling, and (for specific metals) genotoxicity and impaired DNA repair [2]. Hg is a well-established neurotoxicant and nephrotoxicant, and its toxicity is linked to strong binding to thiol/selenol-containing proteins and disruption of cellular redox homeostasis [9,10,11,12]. As is a human carcinogen, and carcinogenic mechanisms involve oxidative stress, altered signaling, and interference with DNA repair, contributing to cancers of the skin and other organs [13,14]. Pb and Cd are systemic toxicants with well-characterized renal and neurological targets, and Cd is also recognized as carcinogenic; these metals likewise converge on oxidative stress-related pathways [2]. Ni and Cr are important from a dermal endpoint standpoint because they are potent skin sensitizers; repeated skin exposure can lead to allergic contact dermatitis through hapten-driven immune activation and inflammatory responses [15,16]. Taken together, these overlapping mechanisms support the biological relevance of evaluating co-occurring metals in cosmetics under dermal exposure scenarios, while acknowledging that toxicity is metal- and species-dependent.

**Figure 1:**
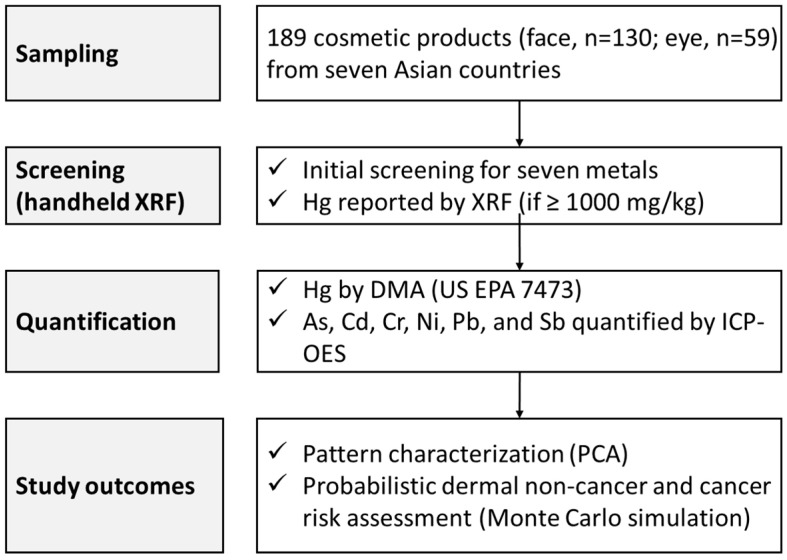
Overall study workflow for sampling, metal screening/quantification (XRF, DMA, ICP-OES), and downstream analyses (PCA and Monte Carlo risk assessment).

Therefore, this study aimed to (i) quantify seven heavy metals (Hg, Pb, As, Cd, Sb, Cr, and Ni) in cosmetic products collected across seven Asian countries (n = 189); (ii) characterize co-occurrence patterns and infer potential contamination signatures using principal component analysis (PCA); and (iii) estimate consumer health risk distributions using probabilistic Monte Carlo simulation, including upper-percentile estimates (e.g., 95th percentile). The overall study workflow is summarized in Figure 1.

## 2. Materials and Methods

### 2.1. Sample Collection

A total of 189 cosmetic products (130 face cosmetics and 59 eye cosmetics) was collected from seven countries (Bangladesh, India, Indonesia, Korea, Malaysia, the Philippines, and Vietnam) between May and June 2022 (Table 1). Products were purchased through offline retail outlets and online platforms in each country to reflect consumer-accessible markets.

### 2.2. Sample Preparation and Analysis

#### 2.2.1. X-Ray Screening of Heavy Metals

All samples were initially screened using a handheld X-ray fluorescence (XRF) analyzer (Delta Professional, OLYMPUS, Tokyo, Japan) without any digestion or other sample preparation. For metals other than Hg, the XRF screening results were used only to determine appropriate dilution factors prior to ICP-based measurement. For Hg, if the XRF-screened concentration exceeded 1000 mg/kg, the XRF value was reported; otherwise, Hg was quantified using a direct mercury analyzer (Section 2.2.2). Since the concentrations of the other elements were below 1000 mg/kg, ICP-OES was used for quantification of heavy metals in cosmetics (Section 2.2.3).

#### 2.2.2. Total Mercury Determination (Direct Mercury Analyzer)

Total mercury (Hg) was quantified using a direct mercury analyzer (DMA-80 evo, Milestone S&T, Sorisole, Italy) based on thermal decomposition, gold amalgamation, and atomic absorption spectrophotometry [17]. This method requires no wet digestion. Approximately 0.01 g of sample was weighed into a nickel/quartz boat and introduced into the analyzer. The drying and decomposition temperatures were set at 200 °C and 850 °C, respectively, with an analysis time of approximately 5 min per sample.

#### 2.2.3. Multi-Element Analysis (Digestion Procedure)

Face cosmetics (~0.15 g) were weighed into Teflon vessels, and 1.2 mL of concentrated nitric acid (HNO_3_, 70%, Sigma-Aldrich, St. Louis, MO, USA) and 0.3 mL of hydrochloric acid (HCl, 37%, Supelco) were added. For eye cosmetics, a smaller aliquot (~0.10 g) was digested with 2.1 mL of HNO_3_ and 0.9 mL of hydrofluoric acid (HF, ≥48%, Sigma-Aldrich) to accommodate the higher inorganic/mineral-pigment content and to maintain an appropriate acid-to-sample ratio for complete digestion under closed-vessel microwave conditions. Microwave digestion procedures followed the manufacturer’s recommended protocols for the MARS 6 system (CEM, Matthews, NC, USA) (“Microwave Digestion of Soaps (Lotion and Foam)” and “Microwave Digestion of Eye Shadow”). The microwave heating program was performed as follows: the temperature was ramped to 190 °C over 15 min and held at this temperature for 15 min. After cooling to room temperature, the digested solutions were filtered through a 0.2 µm PTFE syringe filter (ADVANTEC, Tokyo, Japan) and diluted to 15 mL with deionized water (>18.2 MΩ·cm).

#### 2.2.4. ICP-OES Analysis

Elemental concentrations were determined using Inductively Coupled Plasma Optical Emission Spectrometry (ICPE-9000, Shimadzu, Kyoto, Japan). Operating conditions were: RF power 1.20 kW, plasma gas flow 10.0 L/min, auxiliary gas flow 0.60 L/min, and carrier gas flow 0.70 L/min. Analytical wavelengths (nm) were: As (189.0), Cd (214.4), Cr (267.7), Ni (231.6), Pb (220.4), and Sb (206.8). Calibration curves were prepared using standard solutions (PerkinElmer, Shelton, CT, USA) at concentrations of 0, 10, 25, 50, 100, 250, and 500 µg/L (ppb), with correlation coefficients (R^2^) > 0.999 for all analytes.

### 2.3. Quality Control

Calibration curves for all target elements showed excellent linearity (R^2^ > 0.999). Limits of detection (LODs) were derived from seven replicate measurements at the lowest calibration level. Accuracy was assessed using spike recovery tests at three concentration levels (2, 5, and 10 ppm), yielding recoveries of 88% to 125%. Element-specific LODs and recovery results are summarized in Table S1.

Quality control parameters for Hg (LOD and spike recovery) shown in Table S1 refer to the DMA method. Handheld XRF measurements were used for screening and were reported only when Hg exceeded 1000 mg/kg; these XRF-reported results were not used to derive the QC metrics.

### 2.4. Statistical Analysis

All statistical analyses were performed using R software (version 4.4.3). Detection rate was defined as the proportion of samples with concentrations above the LOD. Different detection rates of criteria were applied depending on the type of analysis. For descriptive analyses of overall metal concentrations and for risk assessment, metals with detection rates ≥50% were retained and values below the LOD were imputed as LOD/√2 (Cr and Ni). For PCA, a detection rate threshold of >40% was applied; Hg, As, Cr, and Ni met this criterion and were included, with values below the LOD similarly imputed as LOD/√2. The normality of data distribution was assessed using the Shapiro–Wilk test.

Principal Component Analysis (PCA) was conducted to identify potential sources of heavy metal contamination in cosmetic products. Prior to analysis, the data were log-transformed to reduce the influence of outliers. PCA was performed using the psych and factoextra packages in R, with Varimax rotation applied to maximize the variance of factor loadings. Principal components were selected based on eigenvalues >1 and a cumulative variance explained of ≥70%. Components with eigenvalues close to 1 were additionally retained when they contributed meaningfully to the cumulative variance and exhibited interpretable loading patterns.

### 2.5. Probabilistic Health Risk Assessment

To estimate the potential health risks posed by heavy metals, the Systemic Exposure Dosage (SED), Hazard Quotient (HQ), and Lifetime Cancer Risk (LCR) were calculated. Unlike deterministic methods that rely on point estimates (mean or max), a probabilistic approach using Monte Carlo simulation was applied to account for the variability in exposure parameters. Probabilistic health risk assessment was performed separately for face and eye cosmetic categories. Based on the number of products included in the analysis, facial creams (n = 111) and mascaras (n = 59) were selected for evaluation.

#### 2.5.1. Systemic Exposure Dosage (SED)

The systemic exposure dosage (SED) estimates the amount of chemicals entering the human body through various exposure pathways and is calculated based on the concentration of heavy metals present in the product under study, the daily amount of product applied [18] (SCSS, 2021), and average body weight of adult females derived from national statistics for each country. Adult female body weight values were obtained from country-specific national data, and the mean of these values was used in the analysis (Table S2) [19,20,21,22,23]. The penetration coefficient for dermal exposure is provided in Table S3. The SED of heavy metals via dermal absorption was calculated using Equation (1):

(1) $$SED(\mathrm{mg}/\mathrm{kg}/\mathrm{day})\;=\;\frac{C\;\times AA\;\times ABS}{BW}\;\times 10^{-3}$$

where *C* is the heavy metal concentration in the cosmetic product (mg/kg), *AA* is the daily amount of product applied (g/day), *ABS* is the dermal absorption factor (unitless), *BW* is the average female body weight (kg), and 10^−3^ is the unit conversion factor from µg to mg, if applicable.

#### 2.5.2. Hazard Quotient (HQ)

The non-carcinogenic risk was assessed by calculating the *HQ* (Equation (2)). An *HQ* value exceeding 1 indicates that adverse systemic health effects are possible.

(2) $$HQ=\frac{SED}{{RfD}_{ABS}}$$

where *RfD_ABS_* is absorbed reference dose (for dermal exposure, mg/kg/day). Its value was calculated from the value of oral reference dose, *RfD_o_*, using the following relationship (3) [24].

(3) $${RfD}_{\mathrm{ABS}}={\mathrm{RfD}}_{o}\;\times \;{ABS}_{\mathrm{GI}}$$

where *ABS_GI_* is the fraction of contaminants absorbed in gastrointestinal tract (dimensionless) in the critical toxicity study [24]. The values of *RfD_o_* [25,26,27,28,29,30] and *ABS_GI_* [31] for most of the heavy metals could be found in the database from the U.S. EPA. For Pb, a RfD has not been established by the U.S. EPA due to the lack of a clearly identifiable exposure threshold [32]. Accordingly, a benchmark dose lower confidence limit for a 10% response (BMDL_10_) of 0.5 µg/kg/day was applied in the analysis [33].

#### 2.5.3. Lifetime Cancer Risk (LCR)

The potential cancer risk associated with exposure to a measured dose of a chemical contaminant was estimated using the lifetime cancer risk (*LCR*), which was calculated using the following Equation (4).

(4) LCR=SED×CSF

Lifetime cancer risk was estimated by multiplying the *SED* (mg/kg/day) by the corresponding cancer slope factor (*CSF*) (mg/kg/day). Probabilistic *LCR* estimates were generated using Monte Carlo simulation, and the resulting distributions were summarized using selected percentiles. For regulatory purposes, the cancer risk considered acceptable by the US EPA is within the range of 1 × 10^–6^ to 1 × 10^–4^. The *CSF* was calculated for Ni, As, Pb, and Cd sulfate by using the respective slope factors of 0.91, 32, 0.0085, and 6.7 (mg/kg/day) [7,34]. This study adopted the updated EPA CSF for inorganic As (31.7 mg/kg/day) released in 2025 [26].

#### 2.5.4. Monte Carlo Simulation

Monte Carlo simulations were conducted using the mc2d package in R, which implements a two-dimensional Monte Carlo framework to explicitly distinguish between variability (V) and uncertainty (U). Input parameters included heavy metal concentrations in products, product usage amounts, dermal absorption factors, body weight and RfD_ABS_; for LCR estimation, CSF was used instead of the RfD_ABS_. The simulations were based on cosmetic exposure scenarios and were conducted using only the detected concentrations of each heavy metal.

Heavy metal concentrations in cosmetic products were modeled as a variability component using lognormal distributions, whereas body weight was modeled as an uncertainty component using a normal distribution. Two-dimensional Monte Carlo simulations were performed with a total of 1,000,000 iterations, consisting of 5000 variability iterations representing heavy metal concentrations and 200 uncertainty iterations representing body weight.

All remaining parameters, including product usage amount, dermal absorption factor, RfD_ABS_ and CSF were treated as point estimates in the simulations. Exposure estimates were summarized using the selected percentiles (50th and 95th) and the maximum value. Detailed information on these variables and their distributions is provided in Tables S2 and S3.

## 3. Results

### 3.1. Determination of Heavy Metals in Cosmetic Samples

Table 2 summarizes detection frequencies and concentrations (median and range) for each metal by country and product category.

#### 3.1.1. Detection Frequency Overview

Cr and Ni were detected in almost all samples, with detection frequencies of 99.2% and 98.5% in face cosmetics (n = 130) and 98.3% and 100% in eye cosmetics (n = 59), respectively. Beyond these ubiquitous elements, face cosmetics showed moderate detection of Hg (52.3%) and lower detection of Pb and As (each 26.9%), followed by Cd (21.5%) and Sb (13.1%). In contrast, eye cosmetics exhibited a markedly higher detection frequency of As (78.0%), while Hg (37.3%) and Sb (23.7%) were detected at intermediate levels; Pb (6.8%) and Cd (5.1%) were detected least frequently.

#### 3.1.2. Face Cosmetics

In face cosmetics, Hg had the highest median concentration (2952 mg/kg; <LOD–67,000 mg/kg), whereas the median concentrations of Cr and Ni were 1.85 mg/kg (<LOD–7.35 mg/kg) and 0.75 mg/kg (<LOD–10.9 mg/kg), respectively. Hg concentrations were particularly elevated in products from the Philippines (median 15,400 mg/kg; max 67,000 mg/kg) and Bangladesh (median 1945 mg/kg; max 24,000 mg/kg), with additional high-end values observed in Malaysia (max 43,700 mg/kg) and India (max 60,400 mg/kg). For the remaining elements, country-specific maxima were observed for Cr in Korea (max 7.35 mg/kg), Ni in India (max 10.9 mg/kg), Cd and Pb in the Philippines (Cd max 24.0 mg/kg; Pb max 38.4 mg/kg), As in Bangladesh (max 14.2 mg/kg), and Sb in India (max 60.8 mg/kg).

#### 3.1.3. Eye Cosmetics

In eye cosmetics, As, Ni, and Cr contributed most to central tendency, with overall median concentrations of 4.13 mg/kg for As (range: ND–29.1 mg/kg), 2.63 mg/kg for Ni (0.48–29.3 mg/kg), and 1.36 mg/kg for Cr (ND–21.8 mg/kg). The highest As and Ni concentrations were observed in products from India (As max 29.1 mg/kg; Ni max 29.3 mg/kg). Cr reached its maximum in Vietnam (max 21.8 mg/kg), followed by India (max 17.7 mg/kg). Sb showed elevated levels in selected samples, with maxima in Bangladesh (38.3 mg/kg) and Vietnam (37.1 mg/kg); notably, Vietnamese eye cosmetics showed a high Sb median (21.6 mg/kg) alongside frequent detection (70%). In contrast, Hg, Cd, and Pb concentrations in eye cosmetics were generally low across all countries (overall medians ND).

### 3.2. Source Characterization Using Principal Component Analysis (PCA)

Correlation analysis indicated moderate-to-strong positive associations among As, Cr, and Ni, whereas Hg showed weak or no correlation with the other metals (Figure S1). Given the high proportion of non-detects for several elements, principal component analysis (PCA) was performed for Hg, As, Cr, and Ni, in order to explore co-occurrence patterns among metals. Two principal components were retained based on eigenvalues and the cumulative proportion of variance explained, accounting for 71.5% of the total variance (PC1: 47.4%; PC2: 24.1%) (Table S4).

**Figure 2:**
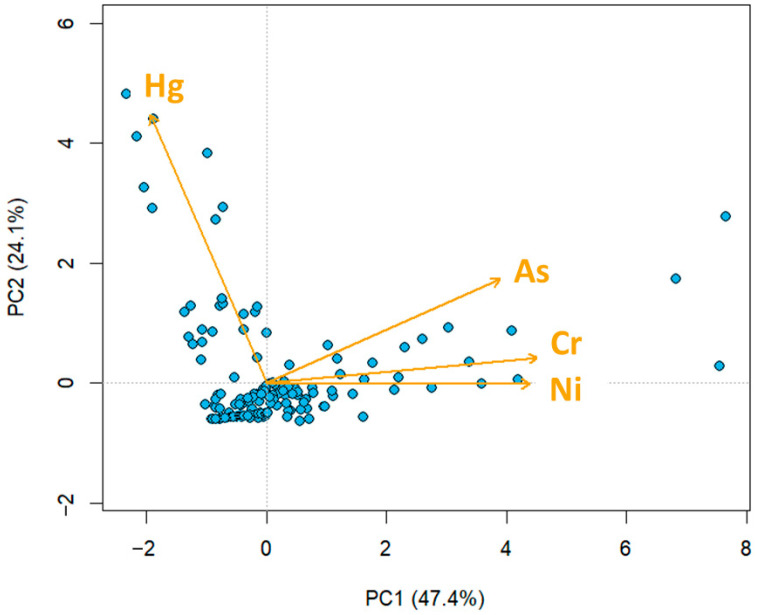
PCA biplot showing sample scores and metal loading vectors (Hg, As, Cr, Ni) in cosmetic products.

PC1 was characterized by high positive loadings of As (0.509), Cr (0.590), and Ni (0.573), indicating a shared pattern of variation among these metals. In contrast, PC2 was dominated by Hg (loading = 0.928), suggesting that Hg varied largely independently of As, Cr, and Ni. Consistently, the PCA biplot showed the Hg vector to be largely orthogonal to the As/Cr/Ni cluster, supporting a distinct contamination signature for Hg relative to the other metals (Figure 2).

### 3.3. Probabilistic Health Risk Assessment (Monte Carlo Simulation)

#### 3.3.1. Non-Carcinogenic Risk Assessment

**Figure 3:**
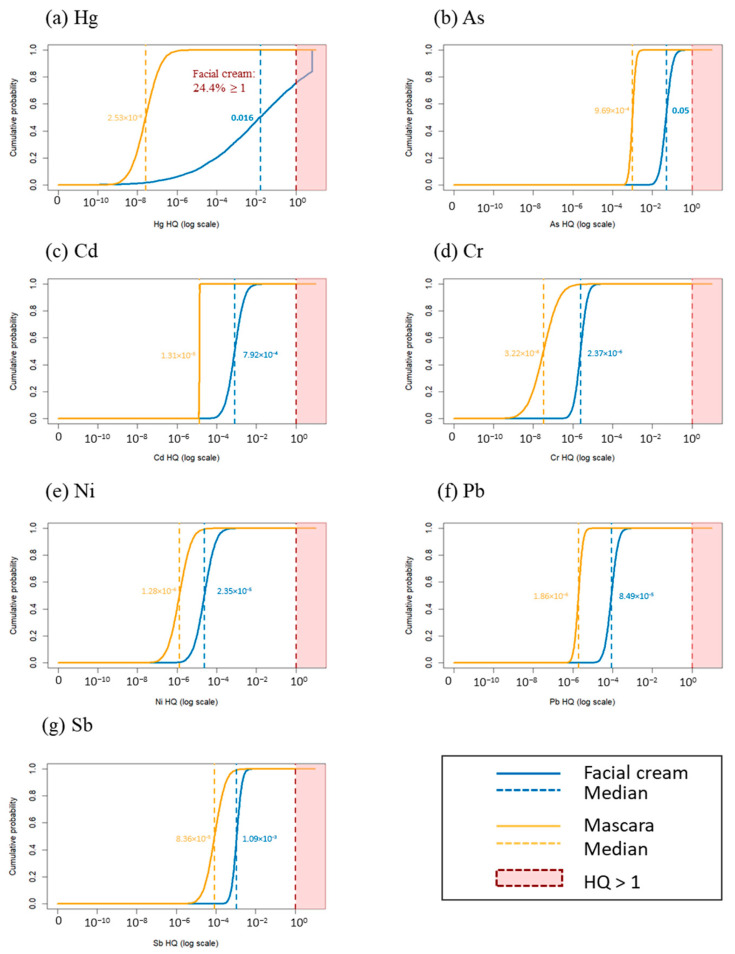
Cumulative probability distributions of hazard quotients (HQs) for dermal exposure to metals in cosmetic products: (**a**) Hg, (**b**) As, (**c**) Cd, (**d**) Cr, (**e**) Ni, (**f**) Pb, and (**g**) Sb. HQ distributions were estimated separately for facial creams (n = 111; blue) and mascaras (n = 55; yellow) using Monte Carlo simulation in R (1,000,000 iterations per product category). The blue and yellow dashed lines indicate the median HQs for facial creams and mascaras, respectively. The red dashed line denotes the HQ threshold of 1, and the shaded area indicates values exceeding this threshold.

To incorporate variability and uncertainty in exposure parameters, systemic exposure dose (SED, mg/kg/day) and hazard quotient (HQ) distributions for dermal exposure were estimated using Monte Carlo simulation for facial creams (n = 111) and mascaras (n = 55) (Figure 3; Table S5).

- Facial creams

Hg showed the highest SED distribution, with a median and 95th percentile of 4.71 × 10^−6^ and 1.90 × 10^−^*^3^*, respectively, followed by As (2.86 × 10^−6^ and 8.14 × 10^−6^). Median SEDs for the other metals were in the 10^−8^–10^−9^ range (Table S5), whereas the median SED for Hg was 4.71 × 10^−6^ (Table S5). In terms of HQ, the median values decreased in the order of As (0.048) > Hg (0.016) > Sb (1.09 × 10^−^*^3^*) > Cd (7.92 × 10^−4^) > Pb (8.49 × 10^−5^) > Ni (2.35 × 10^−5^) > Cr (2.37 × 10^−6^). Notably, the 95th percentile HQ for Hg reached 6.32, with a 24.4% probability of exceeding HQ = 1, whereas no other metal exceeded an HQ of 1.

- Mascaras

Overall SEDs (and corresponding HQs) were one to several orders of magnitude lower than those for facial creams. As exhibited the highest SED and HQ in mascara products (SED: median 5.81 × 10^−8^, 95th 1.02 × 10^−7^; HQ: median 9.69 × 10^−4^, 95th 1.71 × 10^−^*^3^*, max 2.90 × 10^−^*^3^*). For mascara products, all estimated HQs were <1 across the evaluated exposure percentiles (Figure 3; Table S5). For example, Hg had a median HQ of 2.53 × 10^−8^ (95th percentile, 2.88 × 10^−7^).

#### 3.3.2. Carcinogenic Risk Assessment

Probabilistic lifetime cancer risks (LCRs) for As, Cd, Ni, and Pb via dermal exposure were estimated using Monte Carlo simulation (Table 3). As showed the highest LCR among the evaluated metals in both product categories. In facial creams, the median and 95th percentile LCR for As were 9.16 × 10^−5^ and 2.60 × 10^−4^, respectively (max 6.94 × 10^−4^), indicating that the upper tail of the distribution exceeded 10^−4^. For mascara products, LCRs of As were lower than those for face creams, with a median value of 1.86 × 10^−6^, a 95th percentile of 3.27 × 10^−6^, and a maximum of 5.57 × 10^−6^, all remaining below 10^−4^. Under the evaluated exposure scenarios, LCRs for Cd, Ni, and Pb were ≤10^−7^ (Table 3), below the commonly used benchmark levels (e.g., 10^−6^–10^−4^).

## 4. Discussion

In this study, seven metals (Hg, As, Cd, Cr, Ni, Pb, and Sb) were quantified in 189 cosmetic products collected from seven Asian countries. Face cosmetics were characterized by highly heterogeneous Hg levels, including extreme high-end values in a subset of products, whereas eye cosmetics showed comparatively lower Hg levels and were dominated by As, Cr, and Ni. The multivariate structure supported this contrast: Hg behaved largely independently of the other metals, while As, Cr, and Ni co-varied, consistent with a shared pathway related to mineral-based ingredients and pigments. Probabilistic risk assessment further indicated that Hg in facial creams drove the non-carcinogenic risk at the upper tail of exposure, whereas As dominated lifetime cancer risk estimates among the evaluated carcinogens.

### 4.1. Concentrations of Heavy Metals in Cosmetic Products

#### 4.1.1. Mercury (Hg)

The Hg concentrations in face cosmetics showed a dichotomous distribution across the countries. While the median value of Hg from India, Indonesia, Korea, Malaysia, and Vietnam contained <1 mg/kg, extreme outliers were detected in products from Bangladesh, Malaysia, India and the Philippines at 24,000 mg/kg, 43,700 mg/kg, 60,400 mg/kg, and 67,000 mg/kg, respectively. Such extraordinarily high concentrations far exceed levels expected from incidental contamination and strongly suggest that Hg was intentionally incorporated into certain face cosmetic products.

Consistent with our study, a study conducted in Malaysia reported Hg concentrations ranging from 1.81 mg/kg to 838,123 mg/kg in 104 skin whitening products [35]. Similarly, studies conducted in Pakistan have documented high Hg concentrations in skin-lightening creams and beauty creams, with maximum values of 1705 mg/kg [5] and 3047 mg/kg, respectively [36].

In the present study, Hg concentrations in face cosmetics from Indonesia were generally below 1 mg/kg. However, this does not imply the absence of Hg-containing products in the Indonesian market. A previous study reported evaluated Hg levels in skin-lightening creams collected from Indonesian students, ranging from 0.12 to 7834 mg/kg [37]. The maximum Hg concentration reported in skin creams in Bangladesh (0.481 mg/kg) [38] was approximately 5.0 × 10^4^-fold lower than the maximum Hg concentration observed in the present study (24,000 mg/kg). Moreover, a large international analysis of 549 skin-lightening products from 32 countries found that 6.0% contained Hg levels exceeding 1000 mg/kg, with nearly half of these products containing more than 10,000 mg/kg [3]. These discrepancies across studies likely reflect differences in product types, sampling strategies, target populations, and market segments, and further underscore the highly heterogeneous and product-specific occurrence of Hg in face cosmetics.

In Korea, Hg was rarely detected, with concentrations generally low and consistent with previous studies [7,39,40]. One study reported non-detectable levels in body lotions and a maximum of 3.64 mg/kg in sunscreens among 46 face cosmetics from the local market [7]. In addition, two studies analyzing skin products in specific cities reported very low Hg contamination, with maximum concentrations of 0.004 mg/kg in 187 products [39] and 0.014 mg/kg in six products [40], respectively. Although intentional Hg addition cannot be completely ruled out, the detected levels were far lower than the extreme concentrations reported in face cosmetics from other countries, indicating a comparatively lower risk of mercury exposure from Korean products.

Taken together, the highly heterogeneous and product-specific occurrence of Hg suggests that Hg is often intentionally incorporated into certain face cosmetics, particularly skin-lightening products, rather than being present as an incidental contaminant. Hg is often illegally added to skin-lightening products [10] because it inhibits melanin synthesis in epidermal melanocytes by inactivating sulfhydryl enzymes (mercaptans), thereby suppressing tyrosinase activity, a key enzyme in melanin production, and resulting in a lighter skin tone [3,10,41,42]. In addition, Hg is absorbed through the skin, and poisoning may occur following the use of skin-lightening products. Exposure to inorganic mercury can cause kidney and neurological damage, as well as skin rashes, and may pose risks to fetuses when used by women of childbearing age [10]. A study found that students who used mercury-containing skin-lightening cosmetics had higher hair mercury levels compared to non-users [37].

#### 4.1.2. Arsenic (As)

Arsenic exposure, particularly to inorganic forms, has been associated with characteristic skin effects (e.g., pigmentation changes and hyperkeratosis) and increased risks of cancers of the skin, lung, and bladder, with strong evidence primarily from long-term exposure via drinking water and food. Mechanistic studies indicate that arsenic can induce oxidative stress and interfere with DNA repair processes, which are considered relevant pathways in arsenic-related carcinogenesis [13]. In the context of cosmetic use, arsenic present as an impurity may lead to repeated dermal contact; however, dermal uptake is species-dependent and remains less well characterized than ingestion exposure, underscoring the importance of arsenic speciation for toxicity interpretation [2].

In eye cosmetics, total As concentrations had a median of 4.13 mg/kg and a maximum of 29.1 mg/kg (India). Consistent with our findings, a previous study reported As concentrations of 12.14 mg/kg in eyeshadow [43]. Previous studies reported maximum As levels in eye cosmetics (e.g., eye pencils, eyeliners, and eye shadows) ranging from 1.72 to 2.47 mg/kg, which were lower than those observed in the present study [7,44].

In face cosmetics, the maximum As concentration in products from Bangladesh was 14.2 mg/kg, which was higher than values reported for 14 international face foundation powders (up to 0.215 mg/kg) [45] and face creams purchased in Korea (up to 0.67 mg/kg) [7]. In contrast, Ehsan (2024) reported As concentrations in skin-whitening creams from Pakistan, reaching 263 mg/kg [46].

#### 4.1.3. Chromium (Cr)

Cr compounds, including chromium (III) oxide and chromium (III) hydroxide, are intentionally added to cosmetic products as color pigments [2,47], particularly lipsticks, eye shadow, lip makeup, and face powders. In the present study, Cr concentrations in eye cosmetics (ND—21.8 mg/kg) were slightly higher than those reported in Korea (ND—9.17 mg/kg) [7] but lower than levels reported in Iran (30.8–47.0 mg/kg for eye shadow and eye pencil) [48] and Nigeria (25.8–64.3 mg/kg for eyeliner and eye pencil) [49].

In the present study, Cr concentrations in face cosmetics (ND—7.35 mg/kg) were lower than those in eye cosmetics, which showed slightly higher levels but were generally comparable to those reported in previous studies [38,45]. Shomar and Rashkeev (2021) reported levels ranging from 0.039 to 6.741 mg/kg in products produced in Canada, France, Italy, Ireland, and the USA [45]. Another study reported low Cr levels, with five out of six samples below the detection limit and only one sample showing a concentration of 2.82 mg/kg [38]. In addition, skin creams and skin emulsions exhibited low levels of Cr (<2 mg/kg) [7,46,50]. A meta-analysis from Iran reported maximum Cr concentrations in cream products ranging from 29 to 65 mg/kg, which were higher than those observed in the present study [48].

Chromium exists in two valence states, Cr^3+^ and Cr^6+^, both of which can act as potential haptens and contribute to contact allergies [51]. A previous study from Korea analyzed both Cr^3+^ and Cr^6+^ in cosmetic products and found them largely undetectable, except in lip products [7]. In the present study, Cr speciation was not performed.

#### 4.1.4. Nickel (Ni)

Ni is the most common cause of allergic contact dermatitis (ACD) in the general population [52], and cosmetics containing Ni such as mascara and eye shadows might aggravate ACD, particularly in patients with eyelid involvement [53]. In addition, women had 4–10 times higher prevalence of Ni allergy compared with men [54]. Ni is present in coloring agents and can be detected in pigments used across various cosmetic products [55]. In our study, Ni concentrations were higher in eye cosmetics (0.48–29.3 mg/kg) than in face cosmetics, with the highest value observed in products from India (29.3 mg/kg). The range of Ni concentrations observed in the present study was slightly higher than or comparable to ranges reported in previous studies, including 4.4–21.5 mg/kg in eyeliners and eye pencils from Nigeria [49], 0.011–13.3 mg/kg in eyeliners and eye shadows from Korea [7], 3.48–7.97 mg/kg in eyeshadows in online consumer market [56], and 0.021–4.14 mg/kg in eye shadows imported to Italy from China, Italy, and the USA [57]. Some studies have reported even higher concentrations, including up to 73.37 mg/kg in eye shadow from Iran [48] and 42.80 mg/kg in eye pencils from Bangladesh [44].

In the present study, Ni in face cosmetics was detected at lower levels than in eye cosmetics, with a median of 0.75 mg/kg and a maximum of 10.9 mg/kg. Previous studies have also generally reported lower Ni concentrations in face cosmetics (0.46–2.77 mg/kg) compared with eye products [7,56]. In addition, a previous study evaluating Ni in various facial cosmetics, including lotions, foundations, whitening creams, and sunblock creams, reported a maximum concentration of 7.99 mg/kg in sunblock samples [47]. However, in a study from Iran, Ni concentrations in cream products were reported to reach as high as 37.94 mg/kg [48].

#### 4.1.5. Others: Cd, Pb, and Sb

In face cosmetics, Cd, Pb, and Sb were occasionally detected at relatively high concentrations, with Cd reaching 24.0 mg/kg in products from the Philippines, Pb reaching levels of up to 38.4 mg/kg in the Philippines and Sb showing a maximum of 60.8 mg/kg in India. In eye cosmetics, Cd and Pb were rarely detected, with detection rates of 5.1% (maximum 0.36 mg/kg) and 6.8% (maximum 3.20 mg/kg), respectively.

Cd concentrations in the present study were generally low, consistent with previous findings. Earlier studies have reported non-detectable to low Cd levels in body lotions and creams (ND—0.05 mg/kg) and low maximum concentrations in eye cosmetics, including eye shadows (up to 1.27 mg/kg) [7]. Another study analyzing various cosmetic products detected Cd in only 2 out of 19 samples, with a maximum concentration of 0.2 mg/kg [58], while additional studies reported low Cd levels in eye shadows (up to 2.18 mg/kg) and creams (up to 0.08 mg/kg) [48].

Similarly, a large survey of cosmetics reported a median Cd concentration below the detection limit [59], indicating that Cd contamination in cosmetics is generally limited.

While most studies reported Pb concentrations below 1 mg/kg in cosmetics [5,59,60], some products have shown exhibited comparably high levels, reaching up to 8.29 mg/kg in lotions and 3.54 mg/kg in facial cosmetics [7,47], highlighting substantial variability across markets.

In contrast, Sb was more frequently detected in eye cosmetics in the present study (23.7%), with the highest concentration observed in products from Bangladesh (38.3 mg/kg) and Vietnam (37.1 mg/kg). Given that traditional mineral-based eye cosmetics (kohl/surma) have historically incorporated Sb-containing minerals such as stibnite (Sb_2_S_3_), the presence of Sb in some products may reflect mineral pigment inputs rather than inadvertent contamination. However, kohl/surma composition varies widely and has, in many markets, shifted toward Pb-based minerals; therefore, the present data cannot distinguish intentional formulation from contamination without additional information (e.g., ingredients and mineralogical characterization) [61]. However, a previous study analyzing eye cosmetics from Bangladesh reported non-detectable Sb levels [44], suggesting substantial variability in Sb presence across products and regional markets.

#### 4.1.6. Compliance with Applicable Cosmetic Standards for Heavy Metals in Cosmetic Products

For several metals (notably As, Cd, and Sb), the analytical LODs were higher than the most conservative guidance values; therefore, exceedance estimates for these metals should be interpreted as minimum values, as samples reported as non-detect may still exceed guidance values below the LOD. In the exceedance analysis, a sample was classified as exceeding a guidance value only when a quantified concentration was greater than the corresponding threshold; non-detects were conservatively treated as non-exceedances.

Heavy-metal limits for cosmetics differ across jurisdictions, and regulatory frameworks vary in whether they specify numerical impurity limits or focus on prohibiting intentional use while allowing technically unavoidable traces (Table S6) [62,63,64,65]. To enable a consistent cross-country comparison, we primarily evaluated exceedances using the German BVL guidance values (Hg 0.1 mg/kg; As 0.5 mg/kg; Cd 0.1 mg/kg; Sb 0.5 mg/kg; Pb 5 mg/kg for eye products and 2 mg/kg for other products) [64]. For Ni, we applied the Korean MFDS guidance values (35 mg/kg for eye products and 10 mg/kg for other products) because an equivalent BVL guidance value is not available (Table S6) [62,64]. No numerical guidance value was available for total Cr; therefore, Cr was not included in the exceedance-based compliance analysis.

**Figure 4:**
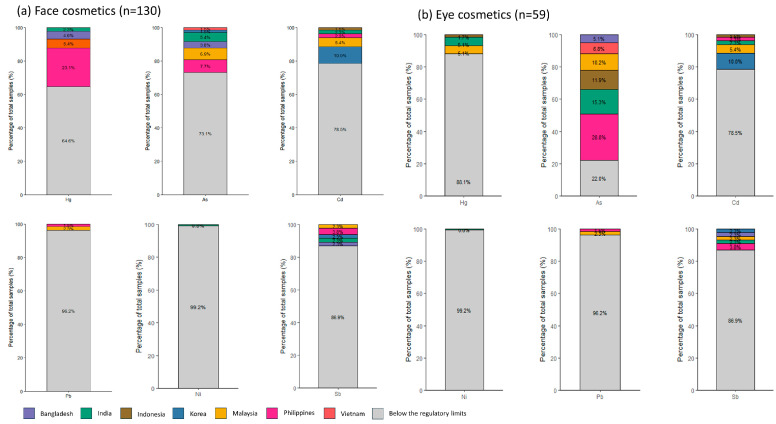
Proportion of cosmetic samples exceeding selected guidance values for metals, evaluated primarily using German BVL guidance values and Korean MFDS guidance values for Ni (Table S6). Results are shown for (**a**) face cosmetics (n = 130) and (**b**) eye cosmetics (n = 59), stratified by metal and country of origin. For metals where analytical LODs exceeded conservative guidance values (notably As, Cd, and Sb), exceedance proportions should be interpreted as minimum estimates.

Figure 4 summarizes the cumulative proportion of samples exceeding the selected guidance values by metal and country, separately for face cosmetics (n = 130) and eye cosmetics (n = 59). Overall, exceedances were driven primarily by Hg in face cosmetics and by As in eye cosmetics, reflecting the markedly different concentration profiles observed between product categories. In contrast, exceedances for Pb and Ni were uncommon under the applied thresholds, while Cd and Sb showed occasional exceedances in specific subsets of products. These patterns indicate that compliance concerns are metal- and product-type-specific and may be sensitive to both the choice of guidance values and analytical detection limits.

### 4.2. Distinct Sources of Heavy Metals in Cosmetics

Principal component analysis indicated two distinct patterns of metal co-occurrence. As, Cr, and Ni loaded together on PC1, suggesting a shared pathway that is plausibly related to mineral-based pigments and raw materials used in color cosmetics. In contrast, Hg dominated PC2 and showed weak association with the pigment-related metals, consistent with a different source pathway. While PCA alone cannot confirm causality, this separation aligns with the interpretation that Hg in a subset of facial products reflects product-specific formulation practices rather than incidental co-contamination with pigment-associated metals.

### 4.3. Risk Assessment

#### 4.3.1. Estimated Exposure to Metals from Cosmetics and Non-Carcinogenic Risk

In this study, the median HQ values were generally higher in face cosmetics (2.37 × 10^−6^ for Cr and 0.048 for As) than in eye cosmetics (3.22 × 10^−8^ for Cr and 9.69 × 10^−4^ for As), indicating higher typical exposure from face products. Notably, the 95th percentile HQ for Hg reached 6.32, with a 24.4% probability of exceeding the threshold HQ of 1. In contrast, previous studies either reported HQs of Hg below 1 [7,45,48] or measured higher Hg levels in skin-lightening products without performing a dermal risk assessment [37,42]. Other studies analyzing heavy metals in cosmetics similarly found HQ values below 1 for dermal exposure across the product types [7,44,48,58].

#### 4.3.2. Estimated Carcinogenic Risk from Cosmetic Use

For carcinogenic risk, As produced the highest LCR estimates in both product categories (Table 3). In facial creams, the As LCR distribution extended into the >10^−4^ range at the upper percentiles, whereas mascara products showed LCRs near the 10^−6^ level and remained well below 10^−4^. Cd, Ni, and Pb yielded LCR estimates ≤10^−7^ under the evaluated dermal exposure assumption (Table 3).

LCR estimates are sensitive to the toxicity values adopted. For inorganic arsenic, the EPA IRIS 2025 assessment reports higher cancer slope factors (e.g., combined CSF 31.7 (mg/kg/day)^−1^ in the low-dose region), which can increase estimated cancer risks relative to earlier values [26]. In addition to As, several metals detected in cosmetics have established or suspected carcinogenic potential. Cd, Cd and Ni compounds are classified as carcinogenic to humans (IARC Group 1); Cr(VI) compounds are also Group 1; and inorganic lead compounds are classified as probably carcinogenic to humans (IARC Group 2A) [66]. Because Cr speciation was not performed in this study (e.g., Cr(VI) vs. Cr(III), Cr(VI))-specific interpretation is not possible, and the results should be interpreted as total Cr.

Mechanistically, these metals can converge on carcinogenesis-relevant pathways, including ROS/oxidative stress, oxidative DNA damage, and interference with DNA repair processes [67,68,69]. Accordingly, co-exposure to multiple metals in cosmetics may involve overlapping biological pathways that are not captured by single-metal toxicity values used in conventional LCR calculations [70].

### 4.4. Implications for Policy and Practice

The observed heterogeneity in metal concentrations across cosmetic products has direct implications for regulatory oversight, manufacturing quality assurance, and public health risk communication. Metals present in cosmetics can remain on the skin and, depending on the element and formulation, may contribute to local effects (e.g., irritation or sensitization) and potential systemic uptake; therefore, repeated dermal contact warrants preventive attention, particularly for products applied frequently and over large surface areas [1].

A risk-based approach to market surveillance is warranted, prioritizing product categories and sales channels that have repeatedly been associated with elevated metal levels, including skin-lightening products and certain eye cosmetics. In particular, routine sampling and enforcement focused on online marketplaces may be important because cross-border e-commerce can facilitate access to non-compliant products; recent studies of online-purchased cosmetics report frequent exceedance of Hg limits and measurable risk metrics [56,71].

Strengthening pre-market notification/registration and product traceability can improve enforcement and enable rapid responses (e.g., recalls and public advisories). Evidence from Malaysia indicates that most mercury-noncompliant skin-whitening products identified in the market were not registered with the national regulator, illustrating how gaps in notification/registration can coincide with higher-risk products [35]. From an industry perspective, consistent implementation of cosmetics GMP frameworks (e.g., ISO 22716), including raw-material control and batch-level documentation, can reduce contamination risks [72].

Given cross-border supply chains and online sales, regional coordination and harmonization of contaminant benchmarks may increase practical effectiveness; ASEAN guidance provides regional contaminant limits for heavy metals (e.g., Pb 10 ppm, As 5 ppm, Hg 0.5 ppm, Cd 0.3 ppm) as one reference point for alignment [63]. Finally, clearer labeling and consumer risk communication—particularly for high-risk categories, and online purchasing channels—can complement surveillance and enforcement efforts, alongside greater platform accountability [73].

### 4.5. Strengths and Limitations

This study provides multi-country data on seven metals in a broad set of face and eye cosmetic products collected across seven Asian markets, enabling direct comparison of concentration distributions by product type and country of origin. The combined use of univariate summaries and PCA helped characterize contrasting co-occurrence patterns, particularly the independence of Hg from pigment-associated metals. In addition, probabilistic risk assessment incorporated parameter uncertainty to describe the upper tail of risk distributions.

Several limitations should be considered. Metal speciation was not performed (e.g., Cr(VI) vs. Cr(III) and As(III) vs. As(V)), limiting toxicity interpretation for elements with species-dependent toxicity. The compliance assessment against highly conservative guidance values is constrained by analytical detection limits for some metals; therefore, exceedance estimates for As, Cd, and Sb should be interpreted as minimum values. In our Monte Carlo simulation, cosmetic use amounts were treated deterministically (SCSS, 2021), whereas metal concentrations were sampled from distributions derived from measured data. Thus, the analysis reflects concentration heterogeneity across sampled products but does not capture inter-individual variability in usage patterns; consequently, overall exposure variability may be underestimated. We note that uneven sample sizes across countries limit the interpretation of direct cross-country comparisons and may also influence pooled concentration distributions because countries contributing more samples can have greater impact on the empirical distribution. Nevertheless, the Monte Carlo-based risk assessment, which considers the distribution of metal concentrations across the pooled dataset, remains valid for characterizing potential risks across the sampled Asian market. Finally, this work focused on dermal exposure and did not address other potentially relevant exposure pathways (e.g., incidental ingestion for lip products) or product authenticity/counterfeit status, which may contribute to extreme concentrations in specific market segments.

## 5. Conclusions

In conclusion, 189 cosmetic products (face and eye) from seven Asian countries were analyzed for seven metals (Hg, As, Cd, Cr, Ni, Pb, and Sb). Facial products exhibited highly heterogeneous Hg concentrations, including extreme values in a subset of samples, whereas eye products were characterized primarily by As, Cr, and Ni. PCA supported this contrast by showing Hg as largely independent of the pigment-associated metals. Probabilistic risk assessment indicated that Hg in facial creams drove non-carcinogenic risk in the upper tail of exposure, while As dominated carcinogenic risk estimates among the evaluated carcinogens.

Taken together, these findings highlight the need for targeted market surveillance and enforcement focusing on high-risk product categories and metal-specific patterns, alongside improved analytical sensitivity when conservative impurity guidance values are used for compliance screening.

## Figures and Tables

**Table 1:** Distribution of collected cosmetic samples by country and product category (n = 189).

Category	Bangladesh	India	Indonesia	Korea	Malaysia	Philippines	Vietnam	Total
**Face cosmetics**	14	9	12	13	14	48	20	130
Facial cream	11	4	11	10	12	45	18	111
Foundation	3	5	-	1	-	1	-	10
**Eye cosmetics**	5	10	8	-	6	20	10	59
Mascara	5	6	8	-	6	20	10	55
Eye shadow	-	4	-	-	-	-	-	4
**Total**	19	19	20	13	20	68	30	189

**Table 2:** Heavy metal concentrations (median, range, and detection rate) in cosmetic products from seven Asian countries (mg/kg).

	Country	Face (n = 130)		Eye (n = 59)	
		DF (%)	Median (Range)	DF (%)	Median (Range)
**Hg**	Bangladesh	9/14 (64.3)	**1945 (ND, 24,000)**	1/5 (20.0)	0.01 (ND, 0.01)
	India	7/9 (77.8)	0.05 (ND, 60,400)	6/10 (60.0)	0.12 (ND, 0.34)
	Indonesia	1/12 (8.33)	ND (ND, 0.01)	2/8 (25.0)	ND (ND, 0.17)
	Korea	2/13 (15.4)	0.01 (ND, 0.01)	NA	NA
	Malaysia	14/14 (100)	**0.11 (0.01, 43,700)**	5/6 (83.3)	0.11 (ND, 0.39)
	Philippines	33/48 (68.8)	**15,400 (ND, 67,000)**	5/20 (25.0)	ND (ND, 0.04)
	Vietnam	2/20 (10.0)	ND (ND, 0.08)	3/10 (30.0)	ND (ND, 0.01)
	Total	**68/130 (52.3)**	**2952 (ND, 67,000)**	22/59 (37.3)	ND (ND, 0.39)
**As**	Bangladesh	5/14 (35.7)	ND (ND, 14.2)	3/5 (60)	3.12 (ND, 3.41)
	India	7/9 (77.8)	4.40 (ND, 9.70)	9/10 (90)	4.53 (ND, 29.1)
	Indonesia	0/12 (0)	ND	7/8 (87.5)	3.94 (ND, 5.91)
	Korea	2/13 (15.4)	ND (ND, 6.23)	NA	NA
	Malaysia	9/14 (64.3)	4.43 (ND, 5.84)	6/6 (100)	4.43 (2.90, 12.3)
	Philippines	10/48 (20.8)	ND (ND, 6.44)	17/20 (85)	4.64 (ND, 11.9)
	Vietnam	2/20 (10)	2.69 (1.24, 4.13)	4/10 (40)	3.34 (ND, 3.68)
	Total	35/130 (26.9)	ND (ND, 14.2)	**46/59 (78.0)**	**4.13 (ND, 29.1)**
**Cd**	Bangladesh	0/14 (0)	ND	0/5 (0)	ND
	India	3/9 (33.3)	ND (ND, 0.31)	2/10 (20.0)	ND (ND, 0.36)
	Indonesia	2/12 (16.7)	ND (ND, 0.28)	0/8 (0)	ND
	Korea	13/13 (100)	0.27 (0.21, 0.74)	NA	NA
	Malaysia	7/14 (50)	0.28 (ND, 0.41)	1/6 (16.7)	ND (ND, 0.34)
	Philippines	3/48 (6.3)	ND (ND, 24.0)	0/20 (0)	ND
	Vietnam	0/20 (0)	ND	0/10 (0)	ND
	Total	28/130 (21.5)	ND (ND, 24.0)	3/59 (5.1)	ND (ND, 0.36)
**Cr**	Bangladesh	13/14 (92.8)	3.50 (ND, 4.88)	4/5 (80)	0.97 (ND, 2.34)
	India	9/9 (100)	1.39 (0.62, 2.97)	10/10 (100)	7.19 (1.75, 17.7)
	Indonesia	12/12 (100)	0.87 (0.53, 5.81)	8/8 (100)	1.19 (1.02, 1.97)
	Korea	13/13 (100)	1.01 (0.68, 7.35)	NA	NA
	Malaysia	14/14 (100)	1.37 (0.28, 2.76)	6/6 (100)	1.21 (1.03, 3.73)
	Philippines	48/48 (100)	1.54 (0.46, 5.43)	19/20 (95.0)	1.22 (ND, 11.3)
	Vietnam	20/20 (100)	2.57 (1.46, 3.27)	10/10 (100)	1.58 (0.78, 21.8)
	Total	129/130 (99.2)	1.85 (ND, 7.35)	58/59 (98.3)	1.36 (ND, 21.8)
**Ni**	Bangladesh	14/14 (100)	1.72 (1.31, 6.84)	5/5 (100)	0.77 (0.60, 9.98)
	India	9/9 (100)	2.61 (0.63, 10.9)	10/10 (100)	9.01 (1.43, 29.3)
	Indonesia	12/12 (100)	0.46 (0.30, 2.78)	8/8 (100)	2.49 (0.64, 9.52)
	Korea	13/13 (100)	0.82 (0.26, 3.22)	NA	NA
	Malaysia	14/14 (100)	0.57 (0.20, 1.25)	6/6 (100)	0.80 (0.64, 1.41)
	Philippines	46/48 (95.8)	0.67 (ND, 3.68)	20/20 (100)	4.26 (0.48, 15.6)
	Vietnam	20/20 (100)	1.10 (0.67, 1.26)	10/10 (100)	1.40 (0.48, 4.54)
	Total	128/130 (98.5)	0.75 (ND, 10.9)	59/59 (100)	2.63 (0.48, 29.3)
**Pb**	Bangladesh	2/14 (14.3)	ND (ND, 1.49)	1/5 (20.0)	ND (ND 3.20)
	India	3/9 (33.3)	ND (ND, 1.36)	0/10 (0)	ND
	Indonesia	3/12 (25.0)	ND (ND, 1.64)	0/8 (0)	ND
	Korea	13/13 (100)	1.11 (0.86, 1.53)	NA	NA
	Malaysia	8/14 (57.1)	1.65 (ND, 4.04)	1/6 (16.7)	ND (ND, 1.70)
	Philippines	5/48 (10.4)	ND (ND, 38.4)	2/20 (10)	ND (ND, 2.53)
	Vietnam	1/20 (5.0)	ND (ND, 1.21)	0/10 (0)	ND
	Total	35/130 (26.9)	ND (ND, 38.4)	4/59 (6.8)	ND (ND, 3.20)
**Sb**	Bangladesh	3/14 (21.4)	ND (ND, 8.99)	2/5 (40)	ND (ND, 38.3)
	India	3/9 (33.3)	ND (ND, 60.8)	0/10 (0)	ND
	Indonesia	0/12 (0)	ND	2/8 (25.0)	ND (ND, 17.1)
	Korea	3/13 (23.1)	ND (ND, 5.29)	NA	NA
	Malaysia	3/14 (21.4)	ND (ND, 2.17)	1/6 (16.7)	ND (ND, 1.80)
	Philippines	5/48 (10.4)	ND (ND, 3.13)	2/20 (10.0)	ND (ND, 15.4)
	Vietnam	0/20 (0)	ND	7/10 (70.0)	21.6 (ND, 37.1)
	Total	17/130 (13.1)	ND (ND, 60.8)	14/59 (23.7)	ND (ND, 38.3)

DF, detected samples/measured samples (%); ND, not detected; NA, not available. The 50th percentile represents the median estimated risk, the 95th percentile represents a high-end exposure scenario, and the maximum corresponds to the highest estimated value observed across all samples.

**Table 3:** Probabilistic lifetime cancer risk (LCR) estimates for As, Cd, Ni, and Pb via dermal absorption from facial creams (n = 111) and mascaras (n = 55) based on Monte Carlo simulation (50th, 95th percentile, and maximum).

	Face Cosmetics (Facial Cream Only, n = 111)			Eye Cosmetics (Mascara Only, n = 55)		
	50th	95th	Max	50th	95th	Max
**As**	9.16 × 10^−5^	2.60 × 10^−4^	6.94 × 10^−4^	1.86 × 10^−6^	3.27 × 10^−6^	5.57 × 10^−6^
**Cd**	1.49 × 10^−8^	3.08 × 10^−7^	3.57 × 10^−7^	1.11 × 10^−9^	1.15 × 10^−9^	1.23 × 10^−9^
**Ni**	1.71 × 10^−8^	9.92 × 10^−8^	6.51 × 10^−7^	9.32 × 10^−10^	6.16 × 10^−9^	4.63 × 10^−8^
**Pb**	3.63 × 10^−10^	1.08 × 10^−9^	3.49 × 10^−9^	7.98 × 10^−12^	1.63 × 10^−11^	3.57 × 10^−11^

## Data Availability

The original contributions presented in this study are included in the article/Supplementary Materials. Further inquiries can be directed to the corresponding author.

## Supplementary Materials

The following supporting information can be downloaded at https://www.mdpi.com/article/10.3390/toxics14020167/s1, Table S1: Analytical parameters and quality control results for heavy metals in cosmetic samples; Table S2: Input parameters and probability distributions used for Monte Carlo simulation; Table S3: Reference doses (RfD_o_, RfD_ABS_), absorption fractions (ABS_GI_, ABS) and cancer risk factors of heavy metals; Figure S1: Pairwise correlations among Hg, As, Cr, and Ni concentrations in cosmetic products; Table S4: PCA results for heavy metals included in the analysis (Hg, As, Cr, and Ni): eigenvalues, explained variance, and component loadings; Table S5: Systemic exposure dose (SED, mg/kg/day) and hazard quotient (HQ) estimates for metals via dermal absorption from facial creams (n = 111) and mascaras (n = 55) based on Monte Carlo simulation; Table S6: Selected regulatory or guidance values for metals in cosmetics (mg/kg) used for exceedance screening in this study.

## References

[1] Borowska S., Brzóska M.M. (2015). Metals in Cosmetics: Implications for Human Health. J. Appl. Toxicol..

[2] Bocca B., Pino A., Alimonti A., Forte G. (2014). Toxic Metals Contained in Cosmetics: A Status Report. Regul. Toxicol. Pharmacol..

[3] Hamann C.R., Boonchai W., Wen L., Sakanashi E.N., Chu C.Y., Hamann K., Hamann C.P., Sinniah K., Hamann D. (2014). Spectrometric Analysis of Mercury Content in 549 Skin-Lightening Products: Is Mercury Toxicity a Hidden Global Health Hazard?. J. Am. Acad. Dermatol..

[4] Chan T.Y.K. (2011). Inorganic Mercury Poisoning Associated with Skin-Lightening Cosmetic Products. Clin. Toxicol..

[5] Akhtar A., Kazi T.G., Afridi H.I., Khan M. (2022). Human Exposure to Toxic Elements through Facial Cosmetic Products: Dermal Risk Assessment. Regul. Toxicol. Pharmacol..

[6] Al-Ashban R.M., Aslam M., Shah A.H. (2004). Kohl (Surma): A Toxic Traditional Eye Cosmetic Study in Saudi Arabia. Public Health.

[7] Lim D.S., Roh T.H., Kim M.K., Kwon Y.C., Choi S.M., Kwack S.J., Kim K.B., Yoon S., Kim H.S., Lee B.M. (2018). Non-Cancer, Cancer, and Dermal Sensitization Risk Assessment of Heavy Metals in Cosmetics. J. Toxicol. Environ. Health—Part A Curr. Issues.

[8] Hostynekjasb J.J., Hinzjb R.S., Lorencejb C.R., Pricejb M., Guyb R.H. (1993). Metals and the Skin. Crit. Rev. Toxicol..

[9] Kang B., Wang J., Guo S., Yang L. (2024). Mercury-Induced Toxicity: Mechanisms, Molecular Pathways, and Gene Regulation. Sci. Total Environ..

[10] Bastiansz A., Ewald J., Saldaña V.R., Santa-Rios A., Basu N. (2022). A Systematic Review of Mercury Exposures from Skin-Lightening Products. Environ. Health Perspect..

[11] Jiang S. (2024). Literature Review of the Potential Harm and Mechanisms of Mercury Poisoning Related to Cosmetics. Am. J. Transl. Res..

[12] Clarkson T.W., Magos L. (2006). The Toxicology of Mercury and Its Chemical Compounds. Crit. Rev. Toxicol..

[13] Speer R.M., Zhou X., Volk L.B., Liu K.J., Hudson L.G. (2023). Arsenic and Cancer: Evidence and Mechanisms. Advances in Pharmacology.

[14] IARC Working Group on the Evaluation of Carcinogenic Risks to Humans (2012). Arsenic, Metals, Fibres, and Dusts.

[15] Adam C., Wohlfarth J., Haußmann M., Sennefelder H., Rodin A., Maler M., Martin S.F., Goebeler M., Schmidt M. (2017). Allergy-Inducing Chromium Compounds Trigger Potent Innate Immune Stimulation Via ROS-Dependent Inflammasome Activation. J. Investig. Dermatol..

[16] Saito M., Arakaki R., Yamada A., Tsunematsu T., Kudo Y., Ishimaru N. (2016). Molecular Mechanisms of Nickel Allergy. Int. J. Mol. Sci..

[17] U.S. Environmental Protection Agency (2007). Method 7473: Mercury in Solids and Solutions by Thermal Decomposition, Amalgamation, and Atomic Absorption Spectrophotometry (SW-846).

[18] Scientific Committee on Consumer Safety (SCCS) (2021). The SCCS Notes of Guidance for the testing of cosmetic ingredients and their safety evaluation, 11th revision, 30–31 March 2021, SCCS/1628/21. Regul. Toxicol. Pharmacol..

[19] Food and Nutrition Research Institute, Department of Science and Technology (2022). Philippine Nutrition 2018–2019 And Figures Expanded National Nutrition Survey (ENNS).

[20] World Health Organization (2025). National Survey on the Risk Factors of Noncommunicable Diseases in Viet Nam, 2021.

[21] NIER (2019). Korean Exposure Factors Handbook.

[22] World Health Organization (2011). Non-Communicable Disease Risk Factor Survey Bangladesh 2010.

[23] ICMR-National Centre for Disease Informatics and Research (2020). National Noncommunicable Disease Monitoring Survey (NNMS) 2017–18.

[24] United States Environmental Protection Agency (US EPA) (2004). Risk Assessment: Guidance for Superfund Volume I: Human Heatlh Evaluation Manual (Part E, Supplemental Guidance for Dermal Risk Assessment) Final.

[25] United States Environmental Protection Agency (US EPA) (1995). Mercuric Chloride (HgCl2) (CASRN 7487-94-7)|IRIS|US EPA.

[26] United States Environmental Protection Agency (US EPA) (2025). IRIS Toxicological Review or Inorganic Arsenic—Summary.

[27] United States Environmental Protection Agency (US EPA) (1999). Cadmium (CASRN 7440-43-9)|IRIS|US EPA.

[28] United States Environmental Protection Agency (US EPA) (1998). Toxicological Review of Trivalent Chromium (CAS No. 16065-83-1).

[29] United States Environmental Protection Agency (US EPA) (1991). Nickel, Soluble Salts; CASRN Various.

[30] United States Environmental Protection Agency (US EPA) (1987). Antimony; CASRN 7440-36-0. Integrated Risk Information System (IRIS) Chemical Assessment Summary.

[31] Agency for Toxic Substances and Disease Registry [ATSDR] (2023). Exposure Dose Guidance for Soil/Sediment Dermal Absorption.

[32] United States Environmental Protection Agency (US EPA) Regional Screening Levels (RSLs)—User’s Guide. https://www.epa.gov/risk/regional-screening-levels-rsls-users-guide?utm.

[33] European Food Safety Authority (EFSA) (2010). Scientific Opinion on Lead in Food. EFSA J..

[34] Office of Environmental Health Hazard Assessment (1999). APPENDIX I-A. List of Compounds and Their Associated Unit Risk Factors (URF).

[35] Wan Mohamed Radzi C.W.J., Nordin F.N.M. (2022). Status of Cosmetic Safety in Malaysia Market: Mercury Contamination in Selected Skin Whitening Products. J. Cosmet. Dermatol..

[36] Bashir H., Ibrahim A.B.M., Ullah H., Anwar S., Rehman T.U., Gul Z., Iqbal B., Abidullah S., Khairy M., Habib M.A. (2025). Heavy Metal in Cosmetics and Its Risk to Future Generation in Remote Area of Azad Jammu and Kashmir Trarkhel District Sudhnoti. Sci. Rep..

[37] Abbas H.H., Sakakibara M., Sera K., Nurgahayu, Andayanie E. (2020). Mercury Exposure and Health Problems of the Students Using Skin-Lightening Cosmetic Products in Makassar, South Sulawesi, Indonesia. Cosmetics.

[38] Alam M.F., Akhter M., Mazumder B., Ferdous A., Hossain M.D., Dafader N.C., Ahmed F.T., Kundu S.K., Taheri T., Atique Ullah A.K.M. (2019). Assessment of Some Heavy Metals in Selected Cosmetics Commonly Used in Bangladesh and Human Health Risk. J. Anal. Sci. Technol..

[39] Lee J.H., Kim J.Y., Park S.G., Lee J.H., Yoon J.H., Kim G.T., Kim H.J. (2019). Comparative Study of Hazardous Heavy Metal Contents by Cosmetic Type. J. Environ. Health Sci..

[40] Koo H., Na Y., Lee S., Min S., Kang J., Jin S.H. (2015). The Analysis of Endocrine Distruptors in Commercial Cosmetics. Anal. Sci. Technol..

[41] Ho Y.B., Abdullah N.H., Hamsan H., Tan E.S.S. (2017). Mercury Contamination in Facial Skin Lightening Creams and Its Health Risks to User. Regul. Toxicol. Pharmacol..

[42] Peregrino C.P., Moreno M.V., Miranda S.V., Rubio A.D., Leal L.O. (2011). Mercury Levels in Locally Manufactured Mexican Skin-Lightening Creams. Int. J. Environ. Res. Public Health.

[43] Abra S., Javed S., Kiran S., Awan H. (2022). Analysis of Lead, Cadmium, and Arsenic in Colored Cosmetics Marketed in Pakistan. Public Health Policy.

[44] Baroi A., Siddique M.A.B., Akbor M.A., Chowdhury F.N., Jamil M.A.R., Uddin M.K., Rahman M.M. (2023). Exposure and Health Risks of Metals in Imported and Local Brands’ Lipsticks and Eye Pencils from Bangladesh. Environ. Sci. Pollut. Res..

[45] Shomar B., Rashkeev S.N. (2021). A Comprehensive Risk Assessment of Toxic Elements in International Brands of Face Foundation Powders. Environ. Res..

[46] Ehsan A., Sultana N., Munir B., Assad N., Ghaffar A., Naeem-ul-Hassan M. (2025). Assessment of Some Heavy Metals for Their Potential Health Implications in the Skin Whitening Creams Available in Pakistani Cosmetic Market. Int. J. Environ. Anal. Chem..

[47] Arshad H., Mehmood M.Z., Shah M.H., Abbasi A.M. (2020). Evaluation of Heavy Metals in Cosmetic Products and Their Health Risk Assessment. Saudi Pharm. J..

[48] Ghaderpoori M., Kamarehie B., Jafari A., Alinejad A.A., Hashempour Y., Saghi M.H., Yousefi M., Oliveri Conti G., Mohammadi A.A., Ghaderpoury A. (2020). Health Risk Assessment of Heavy Metals in Cosmetic Products Sold in Iran: The Monte Carlo Simulation. Environ. Sci. Pollut. Res..

[49] Nnorom I.C., Oji-Nnorom C.G. (2005). Trace Metal Contents of Facial (Make-up) Cosmetics Commonly Used in Nigeria. Afr. J. Biotectnol..

[50] Ayenimo J.G., Yusuf A.M., Adekunle A.S., Makinde O.W. (2010). Heavy Metal Exposure from Personal Care Products. Bull. Environ. Contam. Toxicol..

[51] Thyssen J.P., Johansen J.D., Menné T. (2007). Contact Allergy Epidemics and Their Controls. Contact Dermat..

[52] Ahlström M.G., Thyssen J.P., Wennervaldt M., Menné T., Johansen J.D. (2019). Nickel Allergy and Allergic Contact Dermatitis: A Clinical Review of Immunology, Epidemiology, Exposure, and Treatment. Contact Dermat..

[53] Torres F., Das Graças M., Melo M., Tosti A. (2009). Management of Contact Dermatitis Due to Nickel Allergy: An Update. Clin. Cosmet. Investig. Dermatol..

[54] Chen J.K. (2018). Metal Allergy: From Dermatitis to Implant and Device Failure.

[55] Wang X., Hedberg Y.S., Odnevall I. (2022). Presence of Impurities of Nickel and Cobalt in Facial Cosmetic Pigments and Their Dissolution into Artificial Sweat. Contact Dermat..

[56] Kicińska A., Kowalczyk M. (2025). Health Risks from Heavy Metals in Cosmetic Products Available in the Online Consumer Market. Sci. Rep..

[57] Volpe M.G., Nazzaro M., Coppola R., Rapuano F., Aquino R.P. (2012). Determination and Assessments of Selected Heavy Metals in Eye Shadow Cosmetics from China, Italy, and USA. Microchem. J..

[58] Kilic S., Kilic M., Soylak M. (2011). The Determination of Toxic Metals in Some Traditional Cosmetic Products and Health Risk Assessment. Biol. Trace Element Res..

[59] Hepp N.M., Mindak W.R., Gasper J.W., Thompson C.B., Barrows J.N. (2014). Survey of Cosmetics for Arsenic, Cadmium, Chromium, Cobalt, Lead, Mercury, and Nickel Content. J. Cosmet. Sci..

[60] Saidalavi R., Hashim A., Kishor K.B., Leena P.K., Adake P. (2017). Analysis of Lead and Arsenic in Cosmetics and Assessment of Students Awareness about Cosmetic Toxicity. Int. J. Basic Clin. Pharmacol..

[61] Navarro-Tapia E., Serra-Delgado M., Fernández-López L., Meseguer-Gilabert M., Falcón M., Sebastiani G., Sailer S., Garcia-Algar O., Andreu-Fernández V. (2021). Toxic Elements in Traditional Kohl-Based Eye Cosmetics in Spanish and German Markets. Int. J. Environ. Res. Public Health.

[62] Korea Ministry of Food and Drug Safety (MFDS) Regulation on Cosmetic Safety Standards. https://www.mfds.go.kr/brd/m_211/view.do?seq=14757.

[63] Association of South East Asian Nations (ASEAN) (2019). ASEAN Guidelines on Limits of Contaminants for Cosmetics (Release Version 3.0).

[64] Bund B. (2017). Technically Avoidable Heavy Metal Contents in Cosmetic Products. J. Consum. Prot. Food Saf..

[65] Official Journal of the European Union Regulation (EC) No 1223/2009 of the European Parliament and of the Council ANNEX II LIST OF SUBSTANCES PROHIBITED IN COSMETIC PRODUCTS. https://www.legislation.gov.uk/eur/2009/1223/annex/II/adopted?utm.

[66] International Agency for Research on Cancer (IARC) IARC Monographs on the Identification of Carcinogenic Hazards to Humans. https://monographs.iarc.who.int/agents-classified-by-the-iarc?utm_source=chatgpt.com.

[67] Koedrith P., Seo Y.R. (2011). Advances in Carcinogenic Metal Toxicity and Potential Molecular Markers. Int. J. Mol. Sci..

[68] Silbergeld E.K., Waalkes M., Rice J.M. (2000). Lead as a Carcinogen: Experimental Evidence and Mechanisms of Action. Am. J. Ind. Med..

[69] Luevano J., Damodaran C. (2014). A Review of Molecular Events of Cadmium-Induced Carcinogenesis. J. Environ. Pathol. Toxicol. Oncol..

[70] Jomova K., Alomar S.Y., Nepovimova E., Kuca K., Valko M. (2025). Heavy Metals: Toxicity and Human Health Effects. Arch. Toxicol..

[71] Cadungog D.G.E., Yee J.R.D., Sucgang R.J. (2025). Mercury in Online Skin-Lightening Cosmetics: A Health Risk Assessment of Products from Selected Asian Countries. Food Chem. Toxicol..

[72] International Organization for Standardization (ISO) (2007). Cosmetics-Good Manufacturing Practices (GMP)-Guidelines on Good Manufacturing Practices.

[73] Zero Mercury Working Group (ZMWG) (2023). Online Marketing of Toxic Skin Lighteners: Mercury Cosmetics Marketed as “Solutions” to Dark Skin.

