# Supplementary material for: Concentrations and Probabilistic Health Risks of Seven Metals in Face and Eye Cosmetics Across Seven Asian Countries
Source: Toxics. 2026 Feb 11;14(2):167. doi: 10.3390/toxics14020167 (PMC12944837; doi:10.3390/toxics14020167)
Supplement: Supplementary file 1 [file toxics-14-00167-s001.zip › toxics-4137518-supplementary.pdf]

Supplementary Materials

Concentrations and probabilistic health risks of seven metals in face and eye cosmetics across seven Asian countries

Sohyeon Choi[1], Jae-Hyun Kim[1], Aram Lee[1], Yong-Jun Jeon[1], Won Kim[2], In-Ja Choi[2], Jeongim Park[1*]

[1]Department of Environmental Health Sciences, Soonchunhyang University, Asan, Republic of Korea

[2]Wonjin Institute for Occupational and Environmental Health, Republic of Korea

*Corresponding author

Jeongim Park, PhD

Department of Environmental Health Sciences, Soonchunhyang University

22 Soonchunhyang-ro, Shinchang-myeon, Asan, Chungcheongnam-do, Republic of Korea

Tel: +82-41-530-1269, Fax: +82-41-530-1272, E-mail: jeongim@sch.ac.kr

Table S1. Analytical parameters and quality control results for heavy metals in cosmetic samples

Heavy metal	R^2	LOD (mg/kg)	Recovery (%, average ± SD)		
			Low	Middle	High
Hg	0.999	0.003	96±6	100±3	101±1
As	0.999	1.063	94±6	92±5	93±2
Cd	0.999	0.144	92±1	89±0	88±2
Cr	0.999	0.238	125±2	110±1	105±2
Ni	0.999	0.152	105±4	98±1	96±2
Pb	0.999	0.909	113±4	105±1	98±3
Sb	0.999	1.281	88±9	87±3	88±3

Table S2. Input parameters and probability distributions used for Monte Carlo simulation

**Variables**	**Description**	**Value**	**Unit**	**Probabilistic Distribution**
C	Metal concentrations in the cosmetic products	Varies by metals	mg/kg	Lognormal
AA	Applied amount[a]	Face cream: 1.54<br>Mascara: 0.025	g/day	Point
BW	Adult female body weight	Average: 53.43<br>SD: 4.11<br>Korea: 57.7 (SD: 9.02)[b]<br>Philippines: 56.8[c]<br>Vietnam: 53.0[d]<br>India: 52.6[e]<br>Bangladesh: 49.8[f]<br>Indonesia: not available)	kg	Normal

a (SCSS, 2021)
b (NIER, 2019)
c (DOST-FNRI, 2022)
d (MOH Viet Nam and WHO, 2025)
e (ICMR, 2020)
f (WHO & Ministry of Health and Family Welfare, 2011)

Table S3. Reference doses (RfD_o, RfD_{ABS}), absorption fractions (ABS_{GI}, ABS) and cancer risk factors of heavy metals

Compounds	RfDo (mg/kg/day)	ABS_{GI}	RfD_{ABS} (mg/kg/day)	ABS	CSF (mg/kg/day)
Hg	0.0003[a]	1[g]	0.0003	0.001[h]	-
As	0.00006[b]	1[g]	0.00006	0.03 [g]	32[i]
Cd	0.0005[c]	0.025[g]	0.0000125	0.001[g]	6.7[j]
Cr	1.5[d]	0.013[g]	0.0195	0.001[h]	-
Ni	0.02[e]	0.04[g]	0.0008	0.001[h]	0.91[k]
Pb	-	-	0.0005[l]	0.001[h]	0.0085 [k]
Sb	0.0004[f]	0.15[g]	0.00006	0.001[h]	-

RfD_o Oral reference dose; ABS_{GI} The fraction of contaminant absorbed in gastrointestinal tract; RfD_{ABS} Absorbed reference dose; ABS the dermal absorption fraction for a given metal; CSF Cancer risk factor

a (EPA, 1995); b (EPA, 2025); c (EPA, 1989); d (EPA, 1998); e (EPA, 1991); f (EPA, 1987); g (ATSDR, 2023); h (EPA, 1995); i (EPA, 2025); j; (Lim, 2018) k (OEHHA, 1999); l (EFSA, 2010)

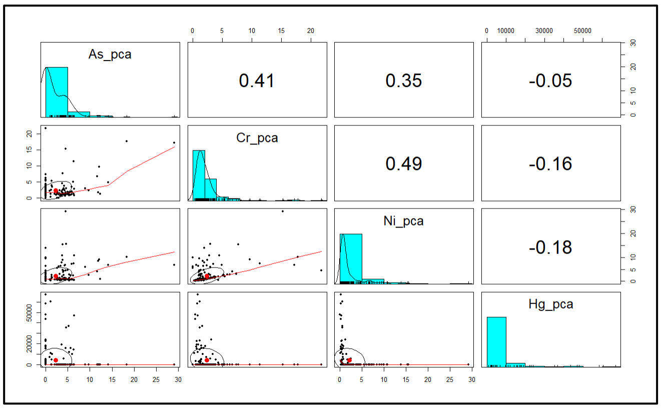

Figure S1. Pairwise correlations among Hg, As, Cr, and Ni concentrations in cosmetic products.

Table S4. PCA results for heavy metals included in the analysis (Hg, As, Cr, and Ni): eigenvalues, explained variance, and component loadings.

Heavy metals	PC1	PC2
Eigen value	1.377	0.982
Total Variance (%)	47.42	24.13
Cumulative Variance (%)	47.42	71.55
As	**0.509**	0.361
Cr	**0.590**	0.088
Ni	**0.573**	-0.003
Hg	-0.253	**0.928**

Table S5. Systemic exposure dose (SED, mg/kg/day) and hazard quotient (HQ) estimates for metals via dermal absorption from facial creams (n = 111) and mascaras (n = 55) based on Monte Carlo simulation

	**Heavy metals**	**SED (mg/kg/day)**			**HQ**		
		**50th**	**95th**	**Max**	**50th**	**95th**	**Max**
**Face cosmetics (Facial cream only, n = 111)**	Hg	4.71×10^{-6}	1.90×10^{-3}	1.90×10^{-3}	0.016	6.32	6.32
	As	2.86×10^{-6}	8.14×10^{-6}	2.17×10^{-5}	0.048	0.136	0.361
	Cd	9.90×10^{-9}	4.60×10^{-8}	1.94×10^{-7}	7.92×10^{-4}	3.68×10^{-3}	0.016
	Cr	4.62×10^{-8}	1.38×10^{-7}	3.88×10^{-7}	2.37×10^{-6}	7.09×10^{-6}	1.99×10^{-5}
	Ni	1.88×10^{-8}	1.09×10^{-7}	5.68×10^{-7}	2.35×10^{-5}	1.36×10^{-4}	7.10×10^{-4}
	Pb	4.25×10^{-8}	1.27×10^{-7}	3.54×10^{-7}	8.49×10^{-5}	2.54×10^{-4}	$7.08. \times 10^{-4}$
	Sb	6.56×10^{-8}	1.53×10^{-7}	3.40×10^{-7}	1.09×10^{-3}	2.55×10^{-3}	$5.66. \times 10^{-3}$
**Eye cosmetics (Mascara only, n = 55)**	Hg	7.59×10^{-12}	8.65×10^{-11}	8.49×10^{-10}	2.53×10^{-8}	2.88×10^{-7}	2.83×10^{-6}
	As	5.81×10^{-8}	1.02×10^{-7}	1.74×10^{-7}	9.69×10^{-4}	1.71×10^{-3}	2.90×10^{-3}
	Cd	1.64×10^{-10}	1.71×10^{-10}	1.79×10^{-10}	1.31×10^{-5}	1.37×10^{-5}	1.43×10^{-5}
	Cr	6.28×10^{-10}	7.55×10^{-9}	7.80×10^{-8}	3.22×10^{-8}	3.87×10^{-7}	4.00×10^{-6}
	Ni	1.02×10^{-9}	6.77×10^{-9}	3.98×10^{-8}	1.28×10^{-6}	8.46×10^{-6}	4.97×10^{-5}
	Pb	9.30×10^{-10}	1.92×10^{-9}	3.78×10^{-9}	1.86×10^{-6}	3.83×10^{-6}	7.56×10^{-6}
	Sb	5.02×10^{-9}	2.81×10^{-8}	1.42×10^{-7}	8.36×10^{-5}	4.69×10^{-4}	2.37×10^{-3}

Table S6. Selected regulatory or guidance values for metals in cosmetics (mg/kg) used for exceedance screening in this study

Heavy metals	Germany[a]	ASEAN[b]	USA[c]	Korea[d]
Hg	0.1	1	Color additive: 1	1
As	0.5	5	Color additive: 3	10
Cd	0.1	5	-	5
Ni	-	-	-	Eye: 35<br>Other: 10
Pb	Eye: 5<br>Others: 2	20	10<br>Color additive: 20	20
Sb	0.5	-	-	10
Cr	-	-	-	-

a BVL (2016)
b ASEAN (2019)
c FDA (2022)
d MFDS (2023)
